# TEM Study of the Microstructure of an Alumina/Al Composite Prepared by Gas-Pressure Infiltration

**DOI:** 10.3390/ma15176112

**Published:** 2022-09-02

**Authors:** Krzysztof Matus, Grzegorz Matula, Mirosława Pawlyta, Jagoda Krzysteczko-Witek, Błażej Tomiczek

**Affiliations:** Faculty of Mechanical Engineering, Silesian University of Technology, Konarskiego 18a Str., 44-100 Gliwice, Poland

**Keywords:** metal matrix composite, powder injection moulding, gas-pressure infiltration

## Abstract

Ceramic injection moulding and gas-pressure infiltration were employed for the manufacturing of alumina/AlSi_10_Mg composites. Porous ceramic preforms were prepared by mixing alumina powder with a multi-binder system and injection moulding the powder polymer slurry. Then, the organic part was removed through a combination of solvent and thermal debinding, and, finally, the materials were sintered at different temperatures. Degrading the binder enabled open canals to form. The sintering process created a porous ceramic material consisting of alumina without any residual carbon content. During infiltration, the liquid metal filled the empty spaces (pores) effectively and formed a three-dimensional network of metal in the ceramic. The microstructure and properties of the manufactured materials were examined using high-resolution transmission electron microscopy, porosimetry, and bending strength testing. Microscopy observations showed that the fabricated composite materials are characterised by a percolation type of microstructure and a lack of unfilled pores. The research confirmed the diversified nature of the connection at the particle–matrix interface. It was observed that the interphase boundary was characterised by the lack of a transition zone between the components or a continuous transition zone, with the thickness not exceeding 30 nm. Thanks to their increased mechanical properties and low density, the obtained composites could be used in the automotive industry as a material for small piston rings and rods, connecting rods, or even gears.

## 1. Introduction

In recent years, there has been an increase in the demand for new lightweight structures that use light metal alloys as the primary construction material. Light metal alloys are increasingly used, especially in the aerospace, transportation, and construction industries. In particular, these materials must have high strength, adequate corrosion resistance, and high safety coefficients. The production of desirable materials is one of the most critical issues in material engineering. An attempt to clarify phenomena that lead to materials with appropriate properties was performed. Hence, it is necessary to investigate the influence of the infiltration parameters of ceramic preforms produced by powder metallurgy on the structure and morphology of precipitates formed in the ceramic–metal composites produced by this method. These parameters have a critical effect on the properties of the finished material and products made from it.

The crucial properties of ceramic–metal composite materials are their resistance to cracking and fracture toughness [1]. The fracture toughness of metal matrix composites is usually limited due to the interfacial debonding between the reinforcement and matrix. Interfacial debonding is mostly caused by interfacial stress induced by differences in the thermal expansion coefficient and elastic modulus between the reinforcement and matrix [2,3,4,5,6,7].

Several mechanisms are responsible for strengthening metal matrix composites. So-called direct strengthening takes place via load transfer from the ductile metallic phase to the hard ceramic [8], while indirect strengthening is due to interactions between the dislocations occurring in the metal–ceramic interface due to their structural mismatch [9]. MMCs are typically produced at elevated temperatures, which, due to the large difference between the thermal expansion coefficients of the ceramic and metallic phases, induce thermal residual stresses. These thermally induced stresses can considerably affect the mechanical properties under external loading [10]. When considering the strengthening mechanism in infiltrated composites, we must take into account the internal load transfer mechanism. If during the deformation of the composite the yield point of the metallic phase is exceeded, its load-bearing capacity is reduced, and the load is transferred to the stiffer and harder ceramic phase. With increasing plastic deformation, the load on the ceramic phase increases steadily, until the ceramics crack or the interfacial surface detaches [11]. In the case of a relatively small strain, the applied compressive stress of both the metallic and ceramic phases undergoes elastic deformation, with a lattice microstrain along the loading direction growing with increasing applied stress at an almost constant rate. A description of the strengthening mechanisms of a composite material with interpenetrating phases is presented in [11]. Various technological attempts were made to increase the mechanical properties of these materials. The heat treatment of fabricated composites has been used to improve the strength properties of metallic matrices while retaining the anti-wear properties of ceramic reinforcements [12]. Over the past few years, the use of liquid metal infiltration process of ceramic preforms for improving composites’ properties has become increasingly popular [13,14,15,16]. Much of the research so far has focused on the effect of manufacturing parameters on the sliding properties and abrasion resistance of the analysed composites [17]. Computer simulation studies of structures and behaviours at the ceramic–metal alloy boundary concluded that the best results are obtained using light metal alloys [14,15,18,19,20]. The highest performance is achieved by the components that are filled with aluminium alloy; for these composites, one of the most popular ceramic phases is alumina [21,22].

One method of manufacturing modern porous materials intended for pressure infiltration is the injection moulding of ceramic powders (known as ceramic injection moulding (CIM). CIM allows for the manufacturing of complex dimensional parts with narrow dimensional tolerances. The mould design and injection parameters highly influence the properties of the finished product. The CIM process usually contains four steps: forming a feedstock of powder–binder mixture, shaping the feedstock using an injection moulding machine, the degradation of the binder, and densification in the sintering process. The powder is mixed with a binder in the injection moulding of ceramic powders. A perfect binder system for CIM must have good flow characteristics, interaction with the powder, debinding, and manufacturing. The optimal binder should have a low contact angle and a low viscosity at the moulding temperature and adhere to the powder during the moulding process. The binder system has to be fully decomposed before sintering. However, the sample must hold the shape during debinding. The binder must be cheap and environmentally friendly for the manufacturing system [23,24,25]. Reports from the literature on the injection moulding of aluminium oxide and binder compositions show that the most commonly used binder is based on polypropylene (PP) [26,27], low-density polyethylene (LDPE) [28,29], and high-density polyethylene (HDPE) [25,30,31], polyethylene glycol (PEG) [32], polystyrene, poly (ethylene-co-) vinyl acetate (EVA) [33], paraffin (PW) [25,26,30], and waxes, e.g., carnauba (CW) [34]. A commonly used surfactant is stearic acid (SA), which is used in all of the compositions mentioned. Recent research suggests that the high strength and toughness of alumina–aluminium alloy composites depend on a trimodal microstructure. Trimodal composites consisting of ultrafine-grained and coarse-grained aluminium and micron-sized ceramic reinforcement particles exhibit combinations of improved strength and ductility. In a trimodal composite, hardness and abrasion resistance reach a very high level due to the ceramic reinforcement, and metal grains with a diameter greater than 500 nm are responsible for an increase in bending strength, while nanometric grains (<100 nm) result in increasing crack propagation energy and a reduction in material brittleness [35,36,37,38,39,40,41]. 

This study tested the hypothesis that composites with the highest bending strength have a trimodal structure. The study also characterised each of the components of the structure and characterised the boundaries between them. Transmission electron microscopies (HRTEM, HRSTEM, SAED, and EDS) were used throughout as the main research techniques.

## 2. Materials and Methods

**Figure 1:**
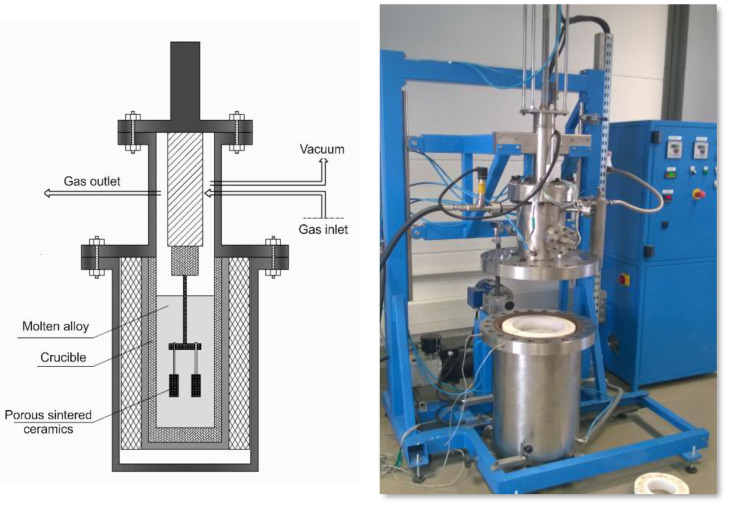
Diagram and view of a device designed for the gas-pressure infiltration of a porous ceramic.

The subjects of the analysis were composite materials obtained using pressure infiltration with an AlSi_10_Mg aluminium alloy of porous ceramic preforms based on sintered alumina powder manufactured with the powder injection moulding method. To produce a ceramic porous preform, coated by stearic acid (SA), Al_2_O_3_ powder was mixed with a multi-component binder using a Zamak Mercator twin-screw extruder machine at a speed of 30 rpm. The binder was made using a mixture of high-density polyethylene (HDPE), polypropylene (PP) and paraffin. This combination of polymers provides the binder with a relatively low viscosity and offers the possibility of using the solvent debinding of paraffin and stearic acid in heptane. Moreover, the thermal debinding of the rest of the skeleton polymers can cause debinding in a large thermal range, which was presented in an earlier article [27,28,29]. The mixing time was dependent on the torque of the binder and feedstock mixture. The powder injection process was performed with a low-pressure Zamak Mercator injection machine. The temperature was experimentally selected from 140 to 180 °C in order to obtain injected parts with high density and high quality. Afterwards, the samples were subjected to a debinding process consisting of solvent and thermal treatments. The degradation and sintering temperatures were selected experimentally with thermogravimetric analysis (TGA) for each binder component separately. The TGA was important to determine the initial and final decomposition temperature of the materials used in this study. Solvent degradation was intended to dissolve the paraffin and stearic acid parts and thereby open pores, allowing deeper access into the material. HDPE was removed during thermal degradation. After the thermal degradation process, samples were sintered for 1 h at various temperatures from 1200 °C to 1600 °C in air at heating rates of 0.5 °C/min. Then, the obtained porous ceramic skeletons were subjected to gas-pressure infiltration with AlSi10Mg alloy under a nitrogen atmosphere at pressures of 1, 2, and 3 MPa. The infiltration process was performed at 800 °C for 180 s using a PTA-8/PrGC2P device produced by the CZYLOK company (Figure 1). 

Observations of the microstructures of both the ceramic preforms and composite materials were made using a Cs-corrected transmission electron microscope S/TEM Titan 80-300 from FEI company (Eindhoven, The Netherlands). Scanning transmission electron microscopy (STEM) and high-resolution transmission electron microscopy (HRTEM) imaging were also used as primary research techniques. The diffraction patterns were obtained with both selected area diffraction (SAED) and Fourier transformations from HRTEM images. Chemical composition examinations were carried out using energy-dispersive spectrometry (EDS). Scanning electron microscopy (SEM) studies were performed with a Zeiss Supra 35 equipped with a field emission gun, EDAX software 6.0, and a TSL OIM EBSD system. Back-scattered electron (BSE) and secondary electron imaging and energy-dispersive X-ray spectroscopy (EDX) were used to determine the correctness of the infiltration method. Structure analysis was performed on the PANalytical X’Pert PRO diffraction system. X-rays were generated at 40 KV and 30 mA using a cobalt target. X-ray qualitative phase analyses of the investigated samples were conducted in the Bragg–Brentano geometry using a PIXcel 3D detector. To determine the mechanical properties, three-point bending tests on a ZWICK mechanical testing machine were performed.

## 3. Results

### 3.1. Microstructure of Porous Sintered Ceramics

**Figure 2:**
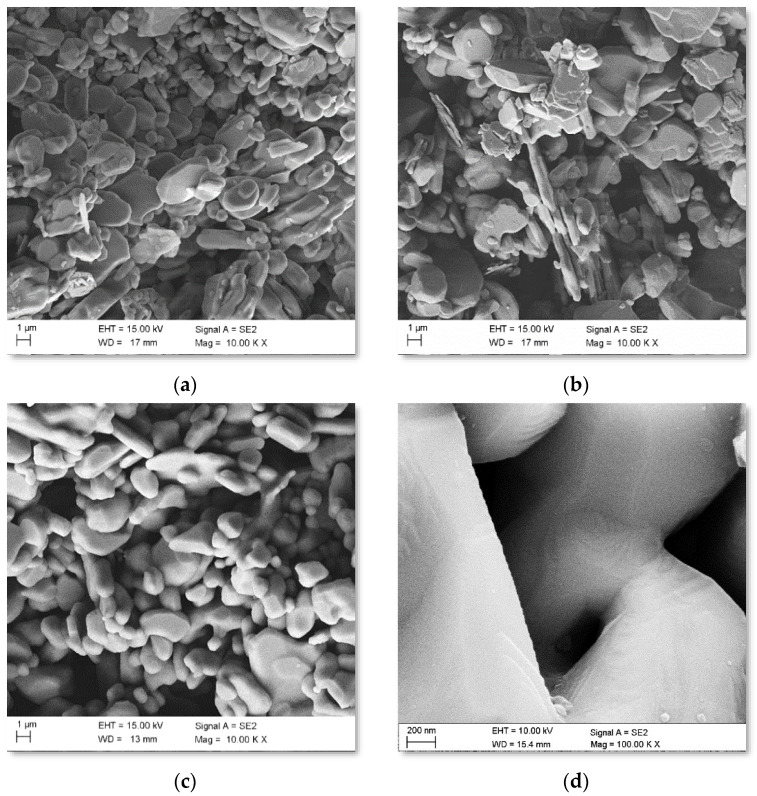
SEM images of fractures in porous ceramic skeletons sintered at (**a**) 1200 °C, (**b**) 1400 °C, and (**c**,**d**) 1600 °C.

Fracture surface images of the porous sintered ceramics obtained using SEM showed no visible defects in the structure in the form of cracks, gas bubbles, or pore clusters that can arise after the degradation of polymeric binders. In particular, relatively small and irregular pores that occurred around the sintered fine ceramic particles (Figure 2a–c) were identified. The diameters of the pores were characterised by a narrow size distribution, and the pore distribution, regardless of the examined area, was homogenous. Comparing the morphology of sintered ceramics at 1200–1600 °C, it can be observed that an increase in the sintering temperature reduces the surface of the particles through their spheroidization and by smoothing the surface and bonding the particles. The analysis of the morphology of the fractures of the ceramic skeletons proved that a sintering temperature of 1600 °C leads to the densification of the powder particles, the formation of so-called necks, and a rounding of the edges of the sintered particles (Figure 2d).

**Figure 3:**
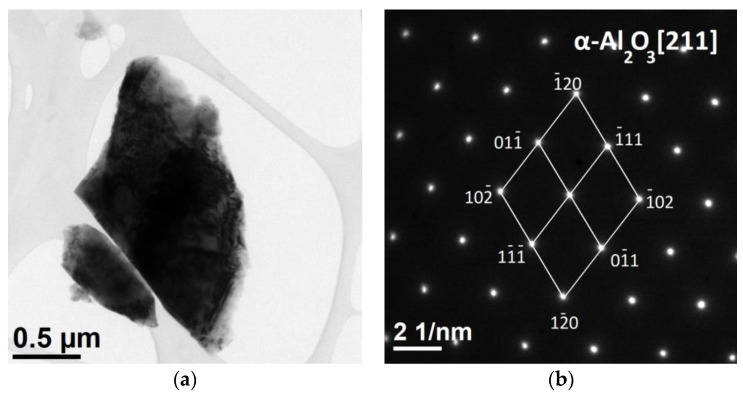
Microstructure of porous ceramic skeletons sintered at 1600 °C. (**a**) TEM-BF; (**b**) SAED.

TEM analyses of ceramics sintered at 1600 °C are shown in Figure 3. An exemplary fragment of the tested material had a size of 2 µm and was characterised by a compact structure (Figure 3a). In contrast, no significant changes were observed in the HAADF images, which indicates a homogeneous chemical composition. The results of the chemical composition analysis confirmed the presence of aluminium and oxygen, the atomic contents of which were 40.2% and 59.8%, respectively, which corresponds to the α-Al_2_O_3_ phase. The results of the SAED diffraction analysis (Figure 3b) confirmed the presence of one phase in the tested material, identified as alumina (α-Al_2_O_3_, hexagonal lattice, space group R-3 c, lattice constants: a = 4.7610 Å, c = 12, 9940 Å, α = 90°, γ = 120°, file no. 000096-100-0018).

### 3.2. Porosity and Density

Based on the results of measurements using the porosimetric method (Table 1), the alumina skeletons sintered at 1600 °C had the highest density of 3.89 g/cm^3^, while the materials sintered at 1200 °C had by the lowest density of 3.79 g/cm^3^. By analysing the apparent density, it can be concluded that, regardless of the test method, i.e., porosimetric or hydrostatic, an increase in the sintering temperature causes an increase in the density of sinters and an increase in the proportion of closed pores, which is typical of high sintering temperatures. The apparent density measurement results were practically the same for both measurement methods and the total porosity was very similar. In addition, an open pore fraction of about 50% at low sintering temperatures was equal to the volume of the binder used during powder injection moulding, proving that the binder also acts as a blowing agent. 

Ceramic sinters intended for infiltration with liquid metals should be characterised by relatively high open porosity and, thus, high values of liquid phase permeability. The permeability is also related to the sintering temperature. As the open porosity decreases, the ability of the liquid phase to pass through the alumina skeleton decreases. The decrease in permeability for the sintered material at 1600 °C was about 12% compared to the skeleton sintered at 1200 °C. Based on the results of the permeability measurement, it was found that the pores and channels in the skeletons had an open structure. 

**Figure 4:**
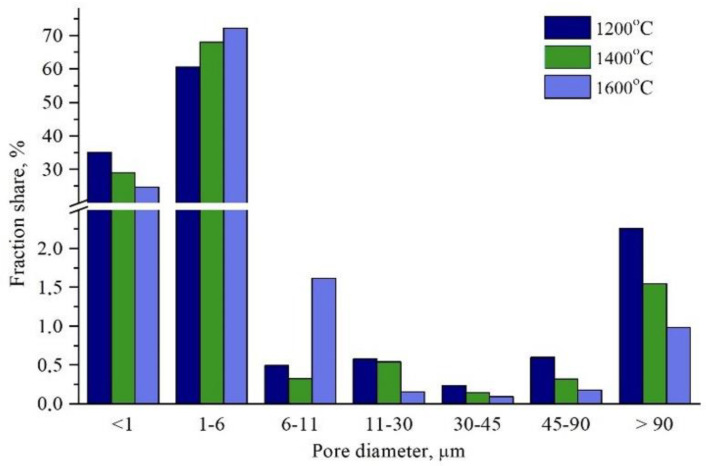
Results of the prosimetry test.

Figure 4 shows that the pore diameters, obtained based on the porosimetric examination of the sinters, were mainly in a range of 1 to 6 μm, and the median diameter increased from 1.3 μm for material sintered at 1200 °C up to 1.4 μm for material sintered at 1600 °C. In the case of the shaped piece sintered at a temperature of 1200 °C, 35% of the macropores had diameters not exceeding 1 μm. The pore diameters, as well as the specific surface area, decreased as the temperature increased from 1200 to 1600 °C, which resulted in the porosity reduction.

The obtained alumina sinters were subjected to pressure infiltration under the set pressures of 1, 2, and 3 MPa. Due to the limited wettability of the ceramic skeleton by the alloy used and the highest proportion of small pores, the spontaneous saturation was unsuccessful. For this reason, we decided to increase the infiltration pressure of the alumina sinters. Density tests of the newly developed composite materials reinforced with alumina skeletons were carried out using a helium pycnometer. The results are presented in Table 2. Based on the obtained results and knowledge of the ceramic fraction share in the sample, the share of the metal phase and the share of non-alloy-filled spaces in the produced composite materials were calculated via the pressure infiltration of porous alumina skeletons. The wettability between aluminium and alumina describes how the metal and ceramic materials interact. Contact angles between solid ceramic and molten metal are used to characterize wetting phenomena. Many researchers measure the wettability between molten aluminium and solid alumina under vacuum [42,43,44]. The differences in the literature data concerning wettability characteristics in the Al/Al_2_O_3_ system result from methodological variances in the measurement procedures and techniques. The overall conclusion is that the contact angles decrease with the increasing temperature. Even though the results are scattered, at temperatures below 900 °C, a wetting angle below 90° is hardly ever observed. A few exceptions concern high chemical purity and a high vacuum regime [45,46], which means that in an infiltration process at a temperature of 800 °C with an initial vacuum of 50 mbar, the liquid aluminium does not wet the surface of the corundum well enough. However, the situation changes during the infiltration process, mainly due to the increase in gas pressure. In that case, aluminium oxide covers the molten aluminium in the casting crucible. When molten alloy enters an alumina porous preform, the oxide layers on the aluminium are broken up into small particles, or it can cover the surface of the preform. The strong metal flow breaks and removes the oxide film, enabling a strong connection on the interface.

The highest average density, 3.32 g/cm^3^, was archived for composite materials reinforced with a ceramic skeleton sintered at 1600 °C and infiltrated under a pressure of 3 MPa, and the lowest composite materials were reinforced with sintered ceramics at 1200 °C and subjected to saturation with liquid metal at a pressure of 1 MPa. The density increased by 42% and 35%, respectively, compared to the density of the alumina skeletons sintered at 1200 and 1600 °C. The density of the composites, compared to that of the sinters, increased as a result of the saturation of the pores with liquid aluminium alloy. The influence of the sintered ceramic phase in a temperature range from 1200 to 1600 °C on the density of the obtained composite materials was also observed. The dependence of the density on the volume of the enhancing fraction was observable and linear. The pressure at which the liquid metal was forced inside the capillaries also had a great influence on the density of the obtained composite materials. It was observed that with the increase in infiltration pressure, the density of the obtained composite materials increased. This trend was caused by the lower proportion of voids and, thus, the higher degree of pores filling with the metal phase. In composite materials prepared with infiltration under a pressure of 1 MPa for a porous sintered skeleton at 1600 °C, the proportion of metal was equal to 44.12%. It increased with the infiltration pressure to 44.74% for 2 MPa and 46.32% for 3 MPa. As a result, the capillary-filling degree improved by 1.41% after infiltration at 2 MPa of pressure and by 4.99% after infiltration at 3 Mpa of pressure, as compared to the saturation of the porous ceramic sample using 1 MPa.

### 3.3. Microstructure and Phase X-ray Diffraction Analysis

**Figure 5:**
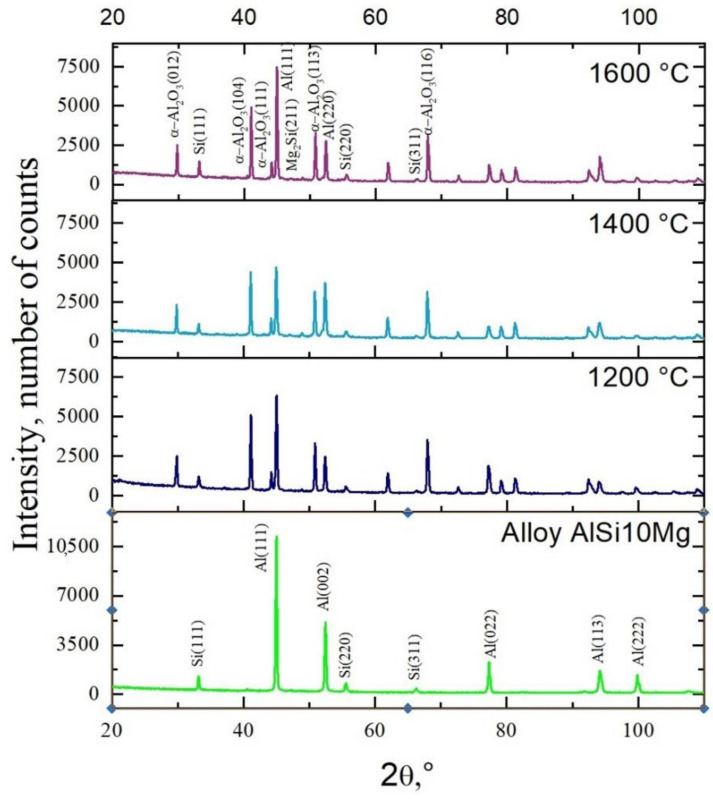
X-ray diffraction analysis results.

As a result of the X-ray analysis (Figure 5), the phase composition of the reinforced ceramic skeleton composites under various conditions was determined. Using methods of qualitative phase analysis, the presence of the α-Al phase (Al, cubic lattice, space group F m-3 m, lattice constants: a = 4.0500 Å, α = 90°, file no. 96-900-846) and the α + e silicon eutectics (Si, cubic lattice, space group F m-3 m, lattice constants: a = 5.4310 Å, α = 90°, file no. 96-210-4749) constituting the matrix of the composite materials were found. In addition, the ceramic phase, α-Al_2_O_3_ (α-Al_2_O_3_, hexagonal lattice, space group R-3 c, lattice constants: a = 4.7610 Å, c = 12.9940 Å, α = 90°, γ = 120°, file No. 000096-100-0018), derived from the reinforcement material in ceramic skeletons and the Mg_2_Si phase, was also identified. Due to the low percentage of the Mg_2_Si (cubic lattice, space group F m-3 m, lattice constants: a = 6.3910 Å, α = 90°, file no. 96-101-0413) phase in the alloy, its identification was based on the diffraction line with the highest relative intensity, 100%. Based on the performed tests, the presence of phases identified based on electron microscopy, along with point analysis of the chemical composition, cannot be excluded due to a low volume fraction beyond the limit of the method detection threshold.

**Figure 6:**
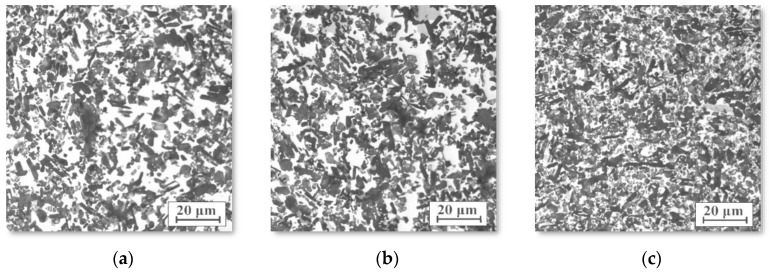
Microstructure of infiltrated composites reinforced with porous ceramics sintered at (**a**) 1200 °C, (**b**) 1400 °C, and (**c**) 1600 °C.

Light microscopy at 200× and 1000× magnification showed that the structures of the composite materials reinforced with alumina sinters were characterised by a fine-grained structure with an even distribution of the reinforcing phase in the alloy matrix (Figure 6). In the photo, the dark areas are ceramic areas and the white areas are the alloy. Regardless of the sintering temperature and infiltration pressure, the materials were characterised by a similar arrangement in the reinforcing phase and a high degree of pores filling with liquid alloy.

**Figure 7:**
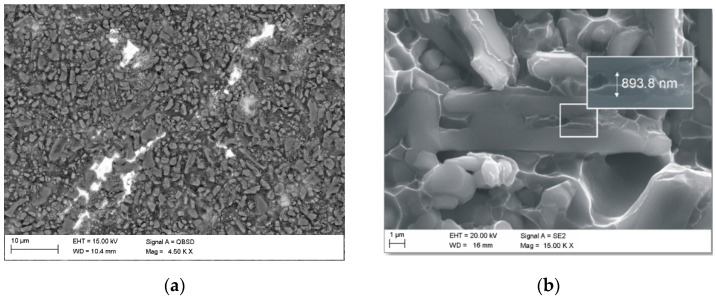
Microstructure (**a**) and fracture surface (**b**) of the infiltrated composites reinforced with porous ceramics sintered at 1600 °C.

SEM imaging characterization showed that the obtained composites had no detectable porosity and demonstrated good filling in the ceramic preform by aluminium alloy (Figure 6). A more detailed analysis found microfiltration in the ceramic matrix (Figure 7), which ensures a good connection between the ceramic and metallic phases and allows for good mechanical properties. The examinations confirmed the regular distribution of sintered alumina particles, the percolation type of the microstructure, and the lack of unfilled pores and reinforcement agglomeration. The ceramic grains retained their shape, which indicates a proper infiltration pressure. The grains were also closely connected with the metallic matrix, which contributes to the achievement of appropriate mechanical properties and indicates a sufficient temperature in the infiltration process.

The correctness of the pressure infiltration with liquid aluminium alloy and the procurement of homogeneous composite material was also confirmed by the results of transmission electron microscopy tests. The example shown in Figure 7 shows a fragment of a composite material (a surface area of approximately 5 μm × 5 μm) produced by sintering a ceramic phase at 1600 °C and then infiltrating it with liquid aluminium alloy at a pressure of 3 MPa. The STEM-HAADF image obtained shows the diversified structure of the composite.

**Figure 8:**
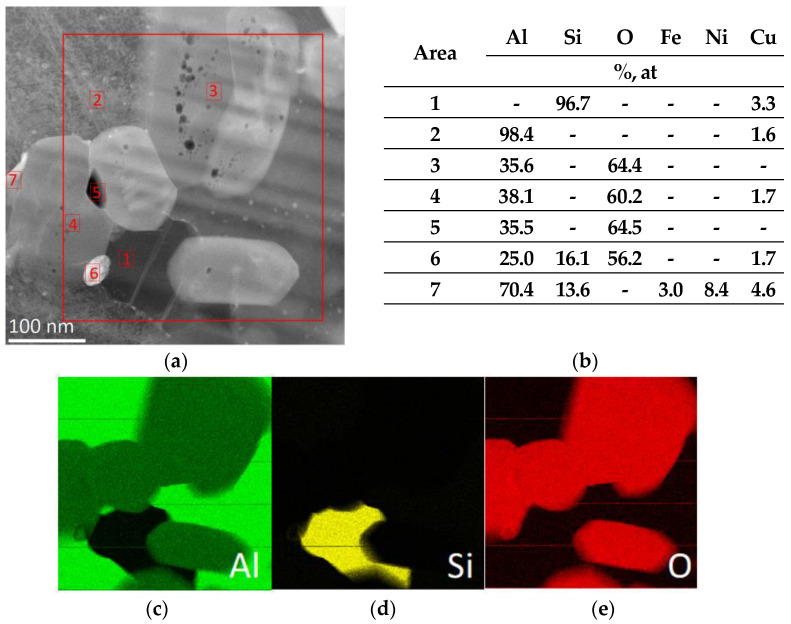
The results of the chemical composition analysis obtained using the characteristic EDS X-ray dispersion spectroscopy (**a**) obtained in the areas indicated in the microscopic image of the composite material produced by sintering the ceramic phase at 1600 °C, infiltrated at a pressure of 3 MP. EDS composition for points 1–7 (**b**), maps of Al (**c**), Si (**d**), and O (**e**).

Figure 8 shows the results of a chemical composition analysis made in points marked with numbers from 1 to 8. The contrast visible in the image indicates a different chemical composition of the composite components. Based on the analysis of the chemical composition, the main components of the composite were identified: silicon (1); aluminium (2); aluminium oxide (3, 4); and phases enriched with nickel, iron, and copper (6–8).

**Figure 9:**
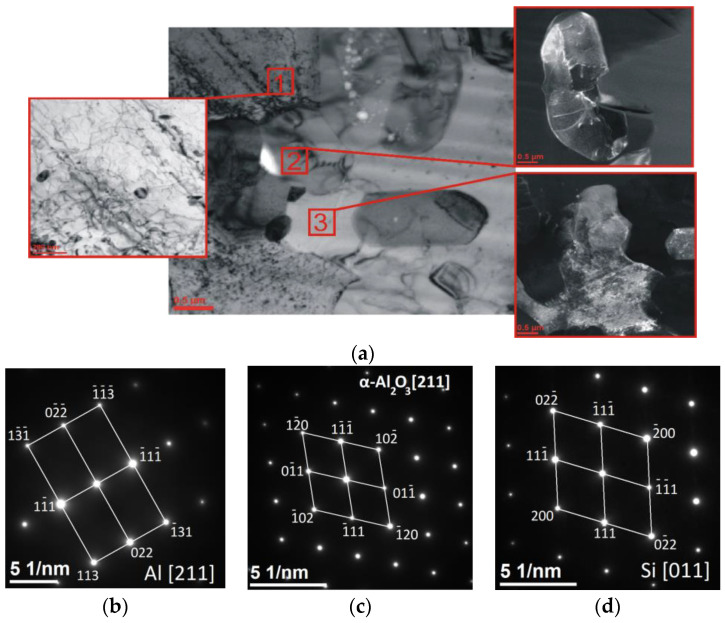
STEM-BF image (**a**) of aluminium (1), alumina (2), and silicon (3) in a composite material, produced by sintering a ceramic phase at 1600 °C, infiltrated at a pressure of 3 MPa. SAED diffraction images of selected grains of (**b**) aluminium, (**c**) alumina, and (**d**) silicon.

Figure 9 shows the following grains: aluminium (Al, cubic lattice, space group F m-3 m, lattice constants: a = 4.0500 Å, α = 90°, file no. 96-900-846), ceramic-phase alumina, (α-Al_2_O_3_, hexagonal lattice, space group R -3 c, lattice constants: a = 4.7610 Å, c = 12.9940 Å, α = 90°, γ = 120, file No. 000096-100-0018), and silicon (Si, cubic lattice, space group F m-3 m, lattice constants: a = 5.4310 Å, α = 90°, file no. 96-210-4749), with resolved diffraction images confirming the presence of these phases.

**Figure 10:**
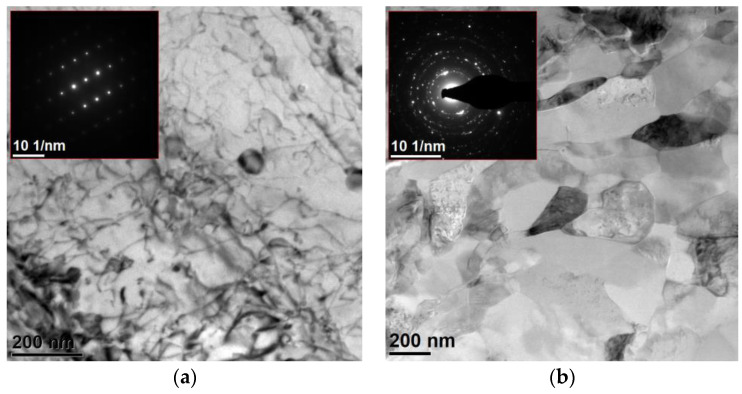
TEM images of (**a**) monocrystalline (**b**) polycrystalline areas in the aluminium alloy matrix.

Further TEM studies provided observations, as expected, of alumina grains a few micrometres in size, with good cohesion at the metal–ceramic interfaces. In the ceramic phase, no cracks were found, which suggests that the compression and infiltration pressures were selected appropriately. In the metallic phase, the presence of cracks and pores was not observed, indicating the correct infiltration process parameters. The observation of the metallic phase confirmed its occurrence in two varieties, and in one of which, grains were micrometre-sized with a high concentration of structural defects, such as dislocations and precipitates (Figure 10a). However, the second type of aluminium grains was characterised by strong polycrystallinity, with grains of 100–200 nm in diameter with a large share of wide-angle boundaries and the elongation of grain parts in the direction of heat removal during crystallisation (Figure 10b). The observations suggest a trimodal structure in the tested composites was achieved with the technological parameters used.

**Figure 11:**
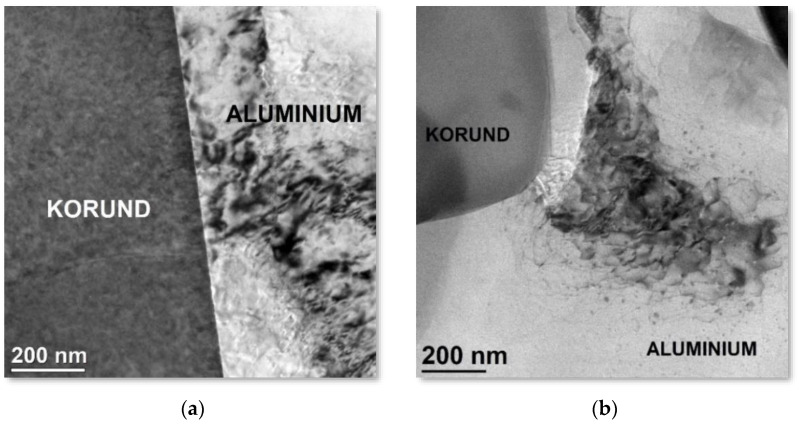
STEM-BF images of (**a**) the interface between alumina and aluminium, (**b**) revealing the influence of the hard phase on the increase in the deterioration of the aluminium structure.

The analysis revealed the presence of two types of precipitation boundaries between the metallic and ceramic phases. The precipitation boundary shown in Figure 11a, between the aluminium alloy and the alumina ceramic, is characterised by a compact structure; there was continuity along the tested joint. There were no voids or delamination on the boundary of the precipitation. The chemical composition analysis did not reveal the presence of additional elements, and a diffusion zone was not observed. In the studied areas, most of the alumina and aluminium grains were well connected with each other. The strongly damaged structure of aluminium caused by the hard and brittle ceramic phase is also visible, which is the source of dislocation (Figure 11b). The highly defected dislocation structure of aluminium is visible in the STEM mode, which resulted from the proportion of diffraction contrast next to the dominant chemical contrast.

**Figure 12:**
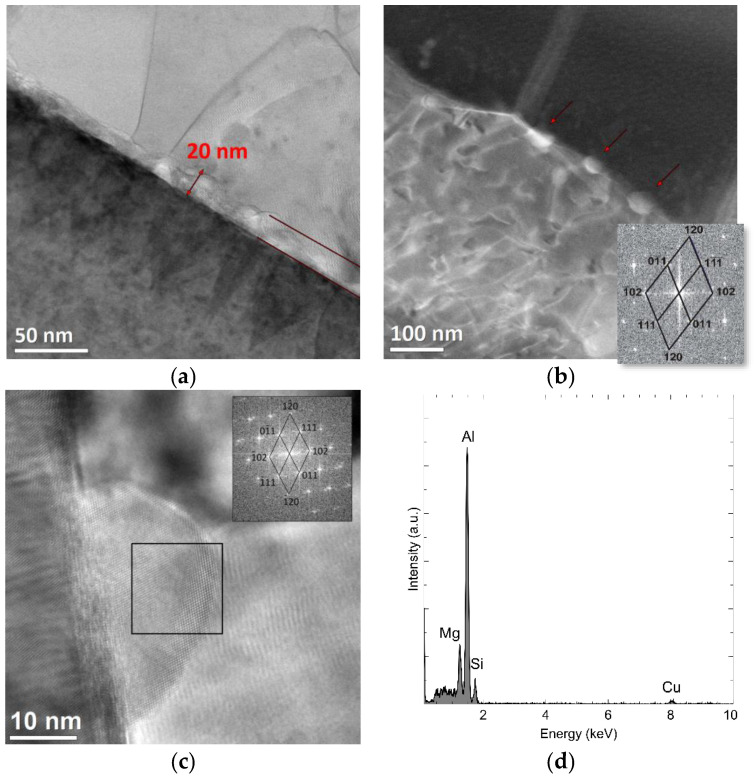
Transition zone between metal and ceramic phase with visible particles. (**a**) STEM-BF image, (**b**) STEM-HAADF image, (**c**) HR TEM image of Al0.45Mg1.5Si precipitation in [211] zone axis with Fourier transform FFT calculated for the marked area, and (**d**) results of the chemical composition analysis obtained using X-ray dispersion spectroscopy.

Figure 12 shows another type of partition boundary: The transition zone between ceramics and metal, about 20 nm thick, is located parallel to the surface of the alumina particles and is distinguished by a different morphology and chemical composition (Figure 12a). In some areas, single precipitates are visible at the border, indicated by arrows in Figure 12b, while in others, they form a continuous layer. The crystalline structure of a single precipitation is visible in Figure 12c. Based on the Fourier transform FFT calculated for the marked fragment, the presence of the Al_0.45_Mg_1.5_Si phase (orthorhombic network, group P n m a, network constants: a = 6.9240 Å, b = 4.1380 Å, c = 7.9620 Å, α = 90°, file no. 96-433-0511) (Figure 12) was confirmed. Figure 12d shows the chemical composition analysis of the same nano-precipitation, containing at least 19% magnesium, 76% aluminium, and 4% silicon. The occurrence of the transition phase between the alumina and the aluminium alloy confirms the diffusion process towards the alumina. The studies using transmission microscopy established that most of the alumina and aluminium grains were well connected with each other in the studied areas.

**Figure 13:**
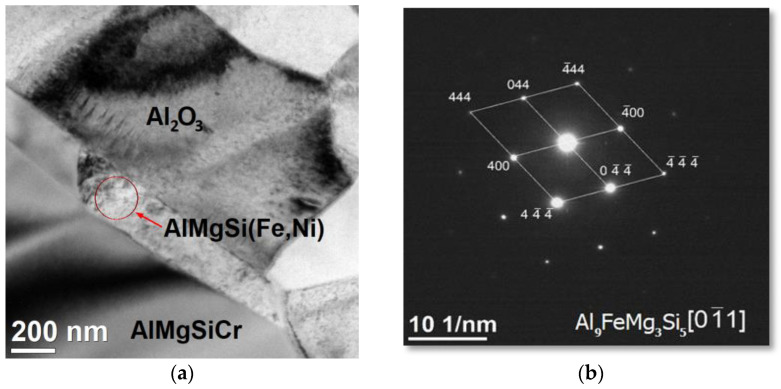
TEM image of the structure of a composite material produced by sintering a ceramic phase at 1600 °C, infiltrated at a pressure of 3 MPa (**a**). The SAED electron diffraction of the precipitation was taken from the area indicated by the arrow (**b**).

There were also three- and multi-component phases (Figure 13). The precipitation in the central part contained about 44% Al, 27% Mg, 21% Si, 3% Fe, and 4% Ni atomically. Based on electron diffraction, they were identified as Al_9_Fe_1_Mg_3_Si_5_ (hexagonal lattice, group P-6 2 m, lattice constants: a = 6.6400, c = 7.9200 Å, α = 90°, β = 120°, file no. 98-009-6905).

**Figure 14:**
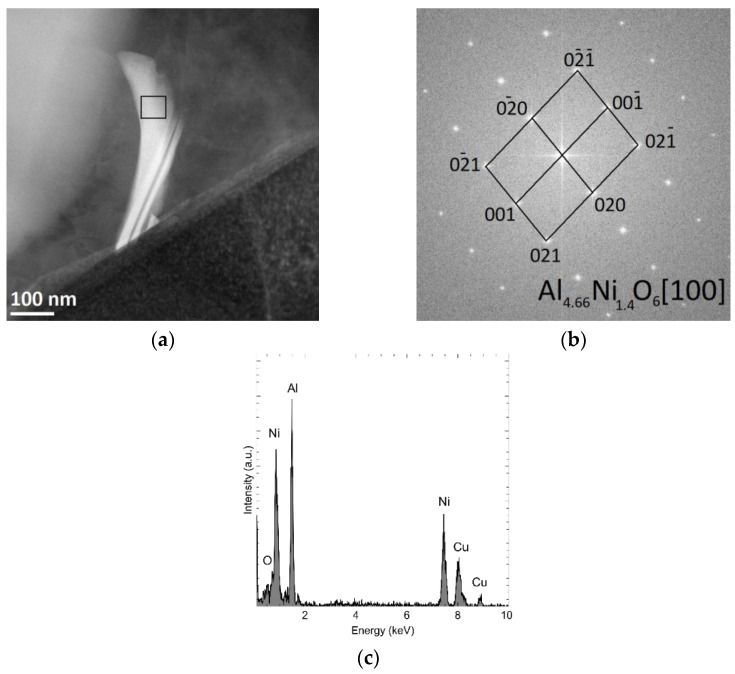
STEM-HAADF image of Al4.66Ni1.4O6 precipitation (**a**), Fourier transform FFT calculated for the HR TEM image taken of the marked area (**b**), and results of the chemical composition analysis obtained using X-ray dispersion spectroscopy (**c**).

In the tested material, grains characterized by a different morphology and chemical composition were also observed. Figure 14 shows the analysis of the chemical composition of the studied precipitation, containing, atomically, about 51% aluminium, 29% nickel, 6% oxygen, and 15% copper (Figure 14c). Using the Fourier transform of the recorded high-resolution TEM image in the area marked in Figure 13a, the investigated phase was identified as Al_4.66_Ni_1.4_O_6_ (orthorhombic lattice, group P bmm, lattice constants: a = 7.6300 Å, c = 2.8900 Å, α = 90°, file no. 2310278) (Figure 14b).

### 3.4. Bending Strength 

The purpose of performing a static bend test was to determine the strength of composite materials and the AlSi_10_Mg alloy constituting the matrix as delivered. Table 3 shows the results of the static bending test on the composite materials produced by the pressure infiltration of porous ceramic sinters. The average bending strength of the AlSi_10_Mg alloy in the delivery condition was 236.5 MPa. In the group of composite materials obtained by infiltrating ceramic skeletons at a pressure of 1 MPa, the highest average flexural strength, 350.3 MPa, was achieved by materials reinforced with a sintered skeleton at 1200 °C, and the lowest average bending strength, 498 MPa, was achieved by composites reinforced with a ceramic phase sintered at a temperature of 1600 °C.

The decreasing tendency in bending strength is related to the increase in the sintering temperature of the reinforcement frames and, thus, the share of the ceramic phase can be observed in all groups of composite materials produced regardless of the infiltration pressure. The higher sintering temperature of ceramic particles results in their better densification, reduction in open porosity, and an increase in the proportion of spaces not filled with alloy (voids). The lower sintering temperature provides a higher proportion of open porosity, enabling better penetration into the shell interior with the alloy at a lower infiltration pressure. There was a noticeable increase in the flexural strength of the composite materials obtained by infiltrating porous skeletons sintered at 1600 °C at a pressure of 3 MPa compared to composites reinforced with ceramic skeletons sintered at 1600 °C and infiltrated at pressures of 1 and 2 MPa. This proves the significant effect of the applied infiltration pressure on the degree of capillary-filling in the skeleton, characterised by a highly compacted structure.

## 4. Discussion

A properly produced alumina skeleton should have a structure of open and interconnected pores and channels, as well as a high permeability value. Observations of the fracture morphology with SEM confirmed that the desired structure was achieved. This was evidenced by the pores formed due to the degradation of the polymeric binder, which was not annihilated during sintering even at a temperature of 1600 °C. Based on the results of density measurements, it was found that the resulting pores and channels had an open structure and, thus, allowed for the easy penetration of the liquid metal alloy during impregnation. It was found that the pore content was from about 50% to about 46% and was dependent on the sintering temperature. The obtained porosity was similar to the results obtained in other works [47,48]. No significant differences in porosity were observed regardless of the test direction. The distribution and shape of the pores did not show any preferential alignment perpendicular to the injection direction, which is typical during uniaxial pressing [49,50]. With an increasing sintering temperature, porosity decreased due to increasing shrinkage. This method of injection moulding aluminium oxide powder and sintering fittings at a temperature of 1200 to 1600 °C resulted in only slight shrinkage in the sinters, amounting to 1% to 5%, respectively. It was found that the apparent density of the porous ceramic scaffolds increased with an increasing proportion of the ceramic phase, which is closely related to increasing the sintering temperature. Due to the very small diameter of the pores and channels, reaching in some cases only up to a dozen nm, the obtained sinters were characterised by a permeability of 1.49 to 1.31 m^2^·10^−15^. The obtained permeability values made it possible to perform gas infiltration in a pressure range from 1 to 3 MPa, eliminating the execution of spontaneous saturation.

The highest density, 3.32 g/cm^3^, was characteristic of a composite with a sintered ceramic skeleton at the maximum temperature, and it was infiltrated under maximum pressure. This is due to the large proportion of the ceramic phase in this composite, which was compacted at a high sintering temperature and saturated with a liquid alloy under high pressure. The lowest density among the tested composites, equal to approx. 3.20 g/cm^3^, was found in materials saturated at a pressure of 1 MPa and reinforced with porous sintered skeletons at a temperature of 1200 °C, in which the ceramic phase content was 40.29%. Typically, alumina-reinforced aluminium composites have a higher density compared to pure alloy [51,52],

Metallographic tests performed with both light and SEM microscopy showed that the structure of the obtained composites was fragmented and homogeneous regardless of the sintering and infiltration conditions. A very low proportion of pores unfilled with aluminium alloy was observed, which proves the correct course of the technological process for producing porous ceramic sinters. Observations of fractures in the composites show that there was a plastically deformed matrix around the reinforcing phase, while at the interface between the ceramic and metal phases, the fracture was brittle [53,54] An analysis of the results of the bending strength tests on the infiltrated composites showed that the highest properties had composites that were sintered at the lowest temperature and then infiltrated, which is also very interesting from an economic point of view. The lower sintering temperature lowers the manufacturing costs and, at the same time, ensures the high open porosity and permeability of the samples. Such prepared skeletons are easier to infiltrate with a liquid alloy, and composites produced in this way are characterised by high properties, regardless of the infiltration pressure. At higher sintering temperatures for ceramic samples, infiltration pressure is more important because the open pores are more constricted, and, hence, greater pressure must be applied to fill them [54,55].

Research on the type of connection between components, carried out with the use of TEM, confirmed the diversified nature of the connection at the particle–matrix interface. It was observed that the precipitation boundary can be characterised by the lack of a transition zone between the components, or a two-phase structure may be formed, which corresponds to the components. The research revealed that, in addition to the joint with a compact, continuous structure, a transition zone may also be created, the thickness of which does not exceed 30 nm, and it contains elements such as magnesium, aluminium, and silicon. The formation of the Al_0.45_Mg_1.5_Si phase on the ceramic–metal interface indicates that a diffusion reaction that took place during the production of composite materials, which was caused by the pressure infiltration of porous ceramic skeletons.

Precipitations in the form of long needles were also observed, including aluminium nickel, and oxygen, as well as multi-component phases containing aluminium, magnesium, silicon, iron, and nickel. Based on the obtained diffraction pattern in Figure 12 and Figure 13, the presence of Al_4.66_Ni_1.4_O_5_ and Al_9_FeMg_3_Si_5_phases was confirmed, respectively.

As the sintering temperature increases, the flexural strength of the composites decreases. It should be assumed that the higher sintering temperature causes the closure of some of the pores and the narrowing of the capillary channels by a more intense diffusion of atoms, and this prevents the liquid metal from reaching these regions during pressure infiltration, especially at low pressure, which results in the lower bending strength of the infiltrated sintered skeletons at a higher temperature.

## 5. Conclusions

In the present work, two modern production technologies were applied and combined, i.e., powder injection moulding and pressure infiltration, which complement each other and enable the production of composites with improved properties. It should be emphasized that this method is intended to produce elements with complex shapes without the need for additional processing. Particular attention should be paid to the polymer binder’s role in the produced composites, which is very important and multifunctional. The selected multi-component binder, with a high proportion of 50%, enables the relatively easy injection moulding of the powder. At the same time, as a pore-forming agent, it provides the desired high porosity of the sinter, which is at least 47% after sintering at the maximum temperature of 1600 °C. The performed sinter tests and infiltrated composites show that the sintering temperature does not have to be high. On the contrary, it should be as low as possible to ensure a high open porosity, which facilitates the filling of pores with liquid aluminium alloy already at a low pressure of 1 MPa. The low porosity of the composites, not exceeding the 2.37% value, proves that the infiltration process was carried out correctly, and the increased infiltration pressure allowed the alumina surface to be wetted with the liquid AlSi_10_Mg alloy, so additional functionalization was not necessary. The occurrence of good wettability was confirmed by structural studies performed with SEM and TEM microscopy, showing the tight connection between the matrix and the reinforcing phase, with frequent precipitations at phase interfaces.

## Figures and Tables

**Table 1:** The results of porosimetric and hydrostatic analyses of sintered ceramic skeletons in a temperature range from 1200 to 1600 °C.

Properties	Sintering Temperature, °C		
	1200	1400	1600
Mercury intrusion volume, cm^3^/g	0.272	0.267	0.223
Specific surface area, m^2^/g	1.09	0.97	0.76
Median pore diameter (vol), μm	1.30	1.37	1.39
Apparent density, g/cm^3^	1.86	1.89	2.10
True density, g/cm^3^	3.78	3.83	3.89
Porosity, %	50.71	50.61	46.49
Permeability, m^2^·10^−15^	1.51	1.37	1.33
Apparent density ^H^, g/cm^3^	1.85	1.89	2.08
Porosity ^H^, %	50.44	50.36	47.79
Open porosity ^H^, %	49.01	47.52	44.45
Closed porosity ^H^, %	1.43	2.84	3.34

^H^ porosity and density measured using the hydrostatic method.

**Table 2:** Results of infiltrated composite density measurements.

Sintering Temperature	1200			1400			1600		
Infiltration pressure, MPa	1	2	3	1	2	3	1	2	3
True density, g/cm^3^	3.20	3.24	3.24	3.24	3.24	3.29	3.26	3.28	3.32
Ceramic phase content, %	49.29	49.29	49.29	49.39	49.39	49.39	53.51	53.51	53.51
Metal phase content, %	49.96	50.71	50.71	50.37	50.6	50.61	44.12	44.74	46.32
Unfulfilled porosity, %	0.75	0	0	0.24	0.01	0	2.37	1.75	0.17

**Table 3:** Results of the bending strength test on the produced composite materials.

Sintering Temperature, °C	1200			1400			1600		
Infiltration pressure, MPa	1	2	3	1	2	3	1	2	3
Bending strength, MPa	498.0	507.4	510.8	470.6	472.0	483.8	350.3	423.6	460.97
Standard deviation	3.01	3.36	2.12	3.25	3.59	3.67	2.31	3.16	1.99

## Data Availability

Not applicable.
